# Online breast-feeding support groups as a community asset in Lebanon after Beirut explosion

**DOI:** 10.1017/S1368980022000295

**Published:** 2022-08

**Authors:** Nabiha Ramadan, Anna Bonmatí-Tomas, Dolors Juvinyà-Canal, Ali Ghaddar

**Affiliations:** 1 Lebanese International University, Mazraa, Beirut 146404, Lebanon; 2 University of Girona, Girona 17003, Spain; 3 Observatory of Public Policies and Health, Beirut, Lebanon

**Keywords:** Breast-feeding, Social media, Community asset, Community resilience, Content analysis, Lactation consultant

## Abstract

**Objective:**

Breast-feeding rates are unsatisfactory in Lebanon. Social media groups could play an important role in promoting breast-feeding in normal conditions and post crisis. The aim of this study is to identify breast-feeding challenges, facilitators and assets and to describe how community assets via social media could build community resilience to pandemic’s and disaster’s effects.

**Design:**

A two-phase qualitative content analysis was performed on posts and comments collected from a Facebook breast-feeding support group. Data were categorised into themes, categories and subcategories.

**Setting:**

Posts and comments retrieved from a Facebook breast-feeding support group in Lebanon during the month of August 2020.

**Participants:**

Group members: mothers who breastfed, breast-feeding mothers and group admins that are lactation consultants.

**Results:**

In phase one, breast-feeding ‘Challenges’ identified were lack of support from peers and family, lack of supportive policies, lack of knowledge and maternal stress related to political instability, COVID-19 and economic crisis. ‘Assets and facilitators’ included community support and donations. In phase two, analysis revealed how assets were being used on social media platform to build community resilience post crisis, through access to social support in challenging times, community engagement, material resources and transformative potential.

**Conclusion:**

Challenges faced during breast-feeding were diminished due to the support and assets received on a Facebook breast-feeding support group, and social media has been shown to be an important community asset implicated in empowering women to breastfeed and to build community resilience in moments of crisis.

Breast-feeding benefits are numerous for mothers and infants. Breastfed infants have lower morbidity and mortality rates^(1)^. Despite its benefits, the rate of breast-feeding remains low worldwide^(2)^. In Lebanon, an upper-middle-income country^(3)^ with growing poverty levels affecting 74 % of the population^(4)^, breast-feeding rates are also disappointing. Even though the initiation rate is high (63·8 %–96 %), exclusive breast-feeding (EBF) drops to 58·3 % in the first month,^(5)^ and the duration of EBF at 6 months varies in different studies where it ranges from 4·1 % to 12·3 %^(5,6)^.

The factors associated with reducing breast-feeding rates in Lebanon^(5,7,8)^ were similar to those found in other countries and included: lack of knowledge about mixed feeding methods and about the importance of breast-feeding, lack of supportive policies, lack of skilled support by the healthcare system and the community, suboptimal maternity leave duration and unsupportive workplace^(9,10)^.

Along with that, in Lebanon, breast-feeding rates are also affected by the socio-economic status, parents’ educational background, cultural beliefs and religion, method of delivery and lacking supportive policies in the healthcare system^(6,7,11)^. Despite the evidence on the important role of International Board Certified Lactation Consultant (IBCLC) on improving EBF duration, their number in Lebanon is very low. For a population of 6 769 146, there are only seventeen IBCLC (the official list is posted on Facebook breast-feeding support group), and they are not part of the national healthcare system^(12)^. All healthcare team members in Lebanon: nutritionists, nurses, midwives, paediatricians practicing lactation consultancy are internationally certified to provide recognised knowledge and support worldwide.

Since the support from peers and from the healthcare system is a weak contributor to breast-feeding in Lebanon^(5)^, mothers have to look for community assets and for other support systems. ‘Community assets’ are defined as the available resources and positive capabilities of the community which improve health status and protect against negative health outcomes^(13)^.Concerning breast-feeding, community assets consist of mothers being in a supportive environment of peers and social network of other women who are breast-feeding or who have breastfed, experiencing similar situations^(14)^. A women-oriented support coming from other women with similar experiences had positive impact and was described as part of ‘community asset’^(14)^.

In the context of health promotion and breast-feeding, and as part of community assets, social media have proved to be an effective tool. Social media platform can provide information to a large number of people very rapidly, it allows interaction, engagement, sharing information and experiences and also provides social support, bypassing all health inequalities in different demographic groups^(15–17)^. Many studies investigating the role of social media in breast-feeding stated that mothers were empowered and encouraged to breastfeed due to the support received^(18,19)^.

Facebook pages targeting breast-feeding can enhance knowledge, provide support and improve breast-feeding intentions^(19)^. In Lebanon, there is no study assessing the role of community support on social media. This type of community asset could play an important role in improving breast-feeding rates in a country where health care and family support are minimal, and where challenges of breast-feeding are numerous. These challenges increased in the last year with the economic hardship and political instability accentuated by the August 2020 explosion of Beirut’s port^(20)^, affecting people’s mental health and challenging breast-feeding and its promotion. Actually, Lebanon is experiencing the most severe economic crisis since the mid nineteenth century, associated with political instability and internal armed conflicts^(21)^. Reported barriers in these critical situations are stress and exhaustion, perception of contaminated milk due to stress and low milk supply in addition to misconceptions about breast-feeding in crisis. In countries like Lebanon, where ‘pre-crisis’ formula milk is common, and where healthcare workers are not knowledgeable or not supportive of breast-feeding especially during ‘conflicts’, its promotion becomes difficult^(22)^.

On the other hand, the COVID-19 pandemic posed additional challenges on the promotion of breast-feeding in Lebanon – as in the rest of the world. Despite the latest recommendations published by the WHO about encouraging breast-feeding in mothers suspected or with confirmed COVID-19, the optimal breast-feeding management in COVID- 19 is still uncertain^(23)^. Other studies have reported that breast-feeding is challenging during the pandemic due to isolation and stress and due to the lack of support from the healthcare providers who shifted their focus on the pandemic^(24)^.

Community resilience aims at fast recovery from disturbing events^(25)^ by community engagement, connectedness between community members, individuals and organisations working to overcome barriers and distress^(26,27)^. As a consequence of the overall situation and the disruption caused among the Lebanese population, the community would build community resilience by creating a support network to cope with these drastic changes at all levels. As a result, it would be important to understand how the community is involved in promoting breast-feeding on social media. The aim of this study is to identify the challenges of breast-feeding among mothers in Lebanon and to explore the role of social media support groups as community assets in the context of crisis after Beirut port’s explosion.

## Materials and method

### Study design

An inductive qualitative content analysis design was proposed to analyse the posts and comments retrieved from the breast-feeding support Facebook group having the largest number of active users in the country. The users of the group are mainly mothers who are breast-feeding or who had breastfed, IBCLC which are mainly group administrators and other health professionals as nurses and psychologists. The text analysis was semantic or manifest content^(28,29)^. Content analysis is a commonly used method to understand communication among people and to gain insight into a certain topic^(30)^.

### Study area

We screened Twitter, Instagram and Facebook to identify the breast-feeding-related pages and groups. There is no account related to breast-feeding in Lebanon on Twitter. On Instagram, we found three pages created by IBCLC, two of them are also found on Facebook. These accounts post activities of the IBCLC owning the page, some informative posts, articles and videos. Followers and members do not interact there.

**Fig. 1:**
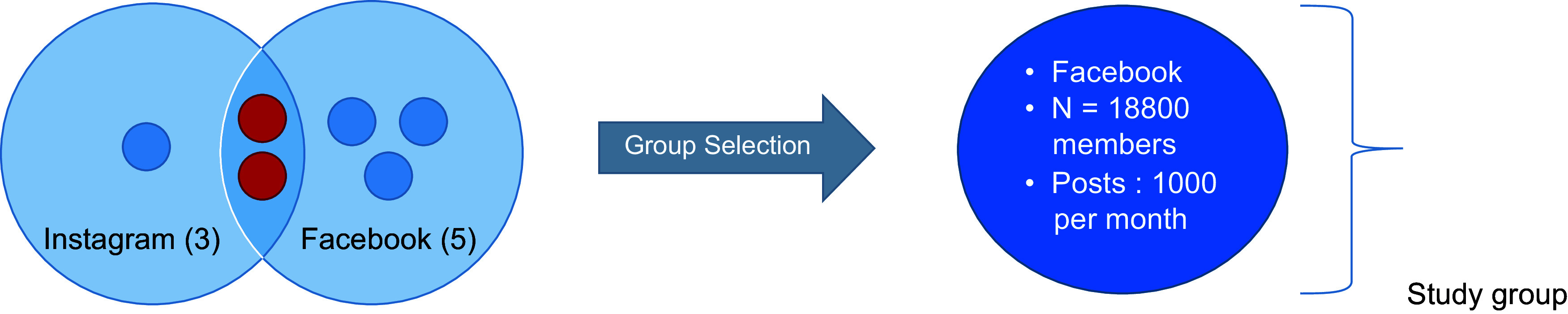
Selection of the unit of analysis

On Facebook, we retrieved five Facebook pages or groups related to breast-feeding in Lebanon including the two also found on Instagram. The third one is ‘La Leche League’ Lebanon-posting announcements of seminars and information related to breast-feeding and health. In the two remaining groups, mainly administrated by lactation consultants, members and admins interact with each other by sharing information, advices and successful stories and concerns, and lactation consultants assist members in their breast-feeding journey. Among these two interactive groups, we chose to analyse the group having the highest number of members and highest daily activity, with 18 800 members to date and users are highly active where posts are on average a little more than 1000/month. The other group has about 8100 members and among these many are members in both groups. The common members write the same posts in both groups that result in duplication. The unit of analysis and its selection is illustrated in Fig. 1.

### Data collection

Posts and comments were retrieved from the Facebook breast-feeding support group during the month of August 2020 after the Beirut port’s explosion. Inclusion criteria were (a) breast-feeding-related posts and comments in English, Arabic and chatting Arabic and (b) posts related to maternal health and infants’ health and feeding schedule. The exclusion criteria were (a) duplicate comments, ‘comments and their corresponding reply’ since these become a form of conversation, and when the comment was written as ‘Up’ in order to keep the post visible and receive more comments to it and (b) photos because these are mainly irrelevant to our study since mothers’ post photos of their kids, photos of breast-feeding gears and breastmilk.

### Data analysis

Data retrieved for analysis included posts and comments in English, Arabic and chatting Arabic. Arabic sentences were translated to English before performing the content analysis. Posts were referred as (P), comments as (C) when we extracted the posts and comments. These posts and comments were written by mothers (M), IBCLC (LC) or other Health Care professional (HC). Analysis was done over two phases: in the first one, barriers of breast-feeding, in addition to facilitators and assets were identified. In the second phase, we focused on identifying how community resilience, in the context of breast-feeding, was built after Beirut’s port explosion.

**Fig. 2:**
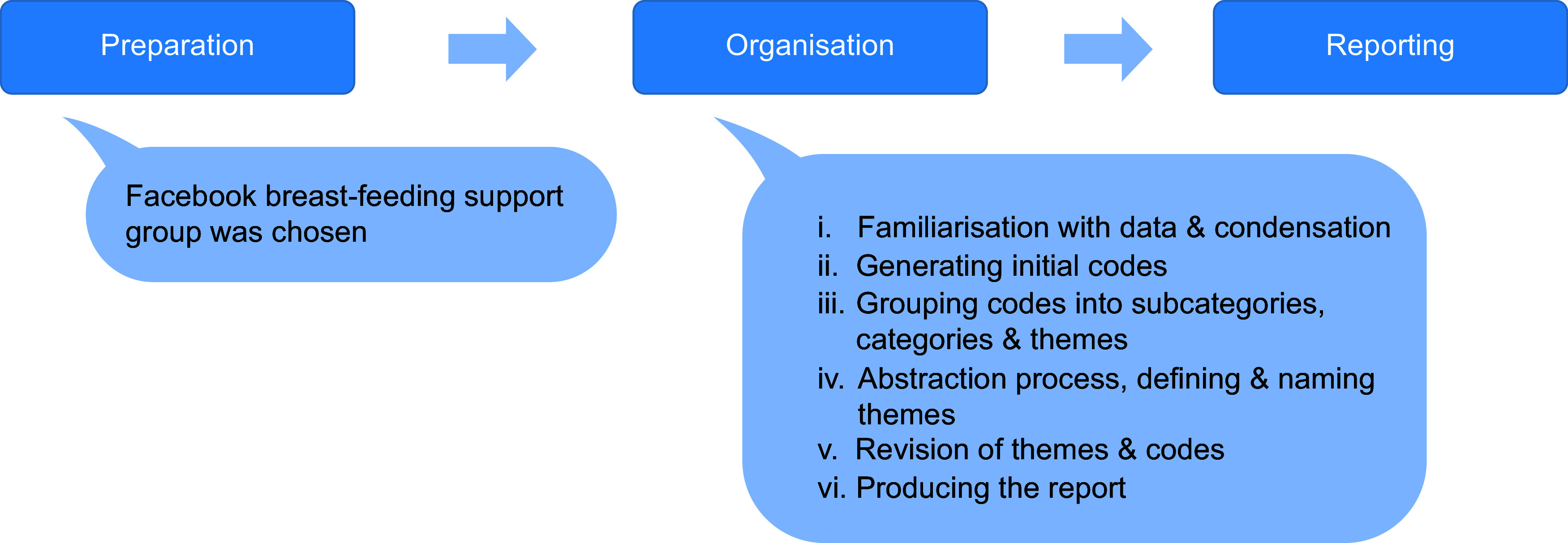
The content analysis: phases I and II

Content analysis in both phases, coding process and categorisation of the text retrieved were performed following the methodology proposed by Elo and Kyngas^(30)^ who described inductive content analysis and recommended a systematic way of organising content into higher order categories to understand a certain phenomenon. The content analysis was done in three steps: preparation, organisation and reporting. In the preparation step, the unit of analysis, the Facebook breast-feeding support group, was chosen. For the organisation step, we followed Braun and Clarke model of thematic analysis that consisted of six phases^(28)^: (i) familiarisation with data and decreasing the sentences’ length while keeping the core meaning, this process is also known as condensation; (ii) grouping sentences with the same meaning or context to generate initial codes; (iii) grouping codes into subcategories, categories and themes; (iv) defining and naming themes, or abstraction process, characterised by the formation of higher order codes and categories done by merging sentences with similar context; (v) revision of themes and codes and (vi) producing the report (Fig. 2). Data collection and the categorisation process were performed in pairs by members of the research team. The Consolidated Criteria for Reporting Qualitative Research checklist was used to ensure the rigor of the research.

## Results

Data collection retrieved a total of 750 posts and 9307 comments with an average of 12·4 comments per post. After using exclusion criteria, 1415 posts and comments were analysed over the two phases. Below are themes, categories and subcategories retrieved in both phases.

### Phase 1

Table 1 describes categories and subcategories related to the two main themes retrieved, which are ‘Challenges and barriers’, and ‘facilitators and assets’ of breast-feeding in Lebanon.

**Table 1:** Themes, categories and subcategories related to breast-feeding barriers and facilitators

Themes	Categories	Subcategories
Challenges and barriers	Lack of support from health care professionals and from peers and family	
	Lack of supportive policies for breast-feeding	
	Maternal emotional stress related to socio-economic and context challenges	– Insecurity and political instability - COVID-19 - Economic crisis
	Misconceptions, suboptimal breast-feeding management and knowledge	
Facilitators and assets	Support from the community	- Supportive messages, information and advices - Sharing successful breast-feeding stories
	Donations and/or selling pumps and gears	

#### Theme 1: challenges and barriers

##### Lack of support from healthcare system, healthcare professionals, peers and family

Results revealed that mothers experienced lack of support from healthcare professionals, mainly from nurses in maternity wards. A common conveyed message from the posts was that nurses relied on provision of formula milk or the encouragement of using it instead of encouraging EBF, as reflected by the view of one mother:

‘Hi Ladies, yesterday I gave birth by C-section. I directly started exclusive breastfeeding. I breastfed the baby every now and then until he slept for 5 hours. When he woke up, the nurse took the baby away gave him formula and now I’m struggling to breastfeed as the baby is not accepting my breast’. (P. M1)

It appeared that paediatricians are not involved in encouraging EBF as well and prescribe formula milk, as reported by a mother:

‘Hello ladies … my son turned 1 month old today so I took him to the pediatrician and his weight is 4200 g and his birth weight was (3770). He told me that my milk is not enough and you should introduce formula milk (1 bottle per day) so that your breast can be filled with milk again … any advice?’.(P. M2)

Another concern was the lack of peer support and family members vouching to use formula milk. The analysis revealed that mothers are asking for advices of how to lower the surrounding negativity towards breast-feeding:

‘Please share your experiences in dealing with wanting to exclusively breastfeeding after delivery and dealing with people (staying at in-laws) telling me maybe we should give formula. The baby stays awake at night and they say he should sleep more, maybe a bottle will help him sleep. He is nursing well, but I have some pain.’ (P.M3)

##### Lack of supportive policies for breast-feeding in Lebanon

Some posts revealed that promotion of breast-feeding in Lebanon was not supported by policies. For instance, women described in their post the unfair low number of maternity leave days and condemned employers for disregarding giving mothers a one-hour break dedicated to breast-feeding or pumping milk during working time:

‘There is no such law. Maternity officially is 70 days as per labor law. Any additional days or any breaks for pumping is internal regulations per company!’ (C. M4)

And for not ensuring a supportive environment for breastfeeding at the workplace:

‘What can I do to maintain my milk supply? I cannot pump at work due to the lack of space’ (P. M2)

##### Maternal emotional stress related to contextual and environmental challenges

The analysis of the posts identified several environmental factors affecting mothers’ ability to breast-feed including the economic crisis and the political instability in the country in addition to the COVID-19 pandemic.

- Insecurity and political instability:

The political internal conflict in Lebanon created challenges to women to practice breast-feeding, as it appeared from some of the inquiry posts about the difficulties experienced to breastfeed while participating in street protests and riots:

‘Is tear gas compatible with breastfeeding?’ (P. M5)

1. COVID-19:

A common concern reflected from several posts shared by breast-feeding moms was about the ability to breastfeed in case of diagnosis with COVID-19. Mothers seemed worried to pass the virus to their newborn. An example is a mother requesting advice about the ability to breastfeed in case of a positive COVID-19 test:

‘I have a terrible fear of getting too sick and not being able to physically breastfeed my EBF 5 months old who doesn’t take bottle or pacifier. Especially in corona times, I am constantly worried and I can’t pump to store milk. Anybody went through corona or a serious illness and how did you manage? What happened to the baby?’ (P. M6) and, ‘Hello mommies. Can a mother with confirmed covid-19 breastfeed her baby?’ (P. M7)

1. Economic crisis:

The economic crisis was an evident barrier to breast-feeding. Mothers in the Facebook groups expressed their concern for their inability to afford buying some items necessary for breast-feeding due to the inflation and currency devaluation, such as pumping machines, breast pads, bottles and other breast-feeding gears. Mothers sought material support in the group and asked about getting breast-feeding gears at a lower price or for free:

‘Hi mommies! Anyone have an electric pump they are willing to donate or sell for an affordable price to a new mother who just gave birth to a tiny baby …’ (P. M8)

##### Misconceptions, suboptimal breast-feeding management and knowledge

Misconceptions about breast-feeding, weak knowledge and ‘know how’ were commonly shared breast-feeding barriers. The most common misconception as it appeared in some posts is the perception of low milk supply, not satisfying their infant’s needs and hunger. In addition to that, mothers were looking for assistance and information about specific breast-feeding conditions like maternal illness and treatment:

‘I am exclusively breastfeeding and I want to know if I have food poisoning, would it affect my baby?’ (P.M9)

Another topic of concern that frequently appeared is dealing with specific conditions experienced during breast-feeding like low milk supply and mastitis:

‘Good evening moms… I’m trying to increase my milk supply. I got a pump but since day 1, milk supply decreased and my breasts got engorged. Is it normal? What should I do?’ (P. M10)

Despite the different topics tackled in posts, they all relate to the lack of knowledge and the need for advice to manage breast-feeding.

#### Theme 2: facilitators and assets

Several facilitators and assets were identified and appeared to increase women’s capabilities to breastfeed by helping them in facing challenges. This theme was divided into mainly two categories and their corresponding subcategories.

##### Support from the community

- Supportive messages, information and advices:

Many supportive messages that encouraged women to breastfeed were evident from multiple cheering and reassuring posts such as

‘Congratulations! be strong dear keep her beside you try to breastfeed as much as you can’ (C. M11).

The encouragement that appears in this post could be reinforcing.

Many mothers who need help, look for advices from other mothers with similar experiences or who are knowledgeable about breast-feeding. It was noted that mothers who posted inquiries asking for advice received many comments with information, advices or referrals.

‘WHATEVER comes out of your breast is the best food for your baby. Contact a lactation consultant it’s still too early and the problem can be fixed easily better now than later’ (C. M12).

Clearly, one part of this comment is encouraging, and the other part includes an advice.

IBCLC and nurses, which are also group members, helped mothers by sharing scientific information or by providing a practical advice such as this post added by a lactation consultant:

‘It’s World Breastfeeding Week! (WHO). Being a parent is the most important job in the world. So it’s important to give parents all the support they need to give their child the best start in life. Breastfeeding is one of them! Support mums to breastfeed anytime, anywhere!’. (P. LC1)

Besides that, some IBCLC in the group also organised live sessions to discuss breast-feeding issues and to provide assistance:

‘Good morning beautiful mommies! …

We’re very excited to announce we will be doing a weekly live session, every Thursday at 9:00 pm. Some weeks we will pick topics and talk about them then answer questions, and some other weeks we’ll just do questions and answers. Tomorrow night, my dear xxxxx xxxx will be here chatting with you. Set your reminders for tomorrow at 9 pm’. (P. LC2)

1. Sharing successful breastfeeding stories:

Sharing successful breast-feeding stories based on personal experience played an encouraging and motivating role to other mothers by showing them that EBF is achievable. The post below is uplifting and also highlights the importance of this breast-feeding support group in the success story of the posting mother:

‘Last Tuesday (18/8/2020) marks the end of our breastfeeding journey. Through all the ups and downs, I am thankful that I was able to breastfeed my son for 2 years and 3 months Thank God. I would like to thank this group and all the mommies who helped me understand all what I need to know throughout this beautiful rewarding journey. …I would like to give a special thanks to (….) who helped me at different times especially when I was down. … I will continue to help other mommies with pleasure”. Thanks w best of luck to all of you supermamas’. (P. M13)

##### Donations and/or selling pumps and gear

Pumping machines, milk bags and breast pads are nowadays required in several breast-feeding cases, when mothers want to return to work or want to go out. Due to the economic situation, many mothers cannot afford buying these as they became very expensive relative to the currency and the salaries. As a result, second-hand markets or low price markets are helpful in this situation. For example, the mother posting here is donating her breast pump as it would help mothers who are unable to buy one:

‘Ladies, I have optimal manual breast pump. I never used it and I would like to give it to a mother who really needs it. I am in south Lebanon. You can send me a personal message.’

And, here is another mother giving breastfeeding essential at a lower price:

‘Hello ladies I have 3 boxes nursing pads, I got them at 12 000 L.L, the old price if anyone interested’ (P. M14).

### Phase 2

Themes and categories related to community resilience are presented in Table 2. In this phase, we focused on how assets were being used on the Facebook breast-feeding support group to build community resilience facing the disruption that occurred. Under the concept of community resilience, we could retrieve four themes and their correspondent categories.

**Table 2:** Themes and subthemes related to breast-feeding after the Beirut 2020 explosion and community resilience

Themes	Categories
Access to social support in challenging times	Communication of scientific information
	Encouraging messages and providing advices
	Emotional support sharing stories, experience, feelings, attitudes and views, facing a challenging situation)
Community engagement	Volunteering that draws people together to work for the benefit of others
	Lactation consultant and psychologists providing their services to the community
Material resources	Milk donations, breast-feeding gears giveaways and a reduced price market
Transformative potential	Community network and cooperation with other organisations

#### Theme 1: access to social support in challenging times

Social support was exhibited by providing supportive and encouraging messages, by giving emotional support and also by providing scientific information.

##### Communication of scientific information

The concerns shared by many mothers about the ability to breastfeed in crisis situations and disturbance due to stress were alleviated by sharing scientific information that eliminated misconceptions. IBCLC answered traumatised mothers concerned about the eligibility to breastfeed post trauma (post Beirut’s port’s explosion):

‘…. The only thing that might be affected is the let down. you might have a delayed let down due to the cortisol secondary to stress. Otherwise, your milk is perfect in terms of quality and quantity’ (C. LC3)

As mentioned in phase 1, IBCLC being part of the group, help mothers by providing trustworthy information as it shows in this comment, replying to a mothers asking about the safety of breast-feeding during COVID-19.

‘Another source that says you should keep breastfeeding, even if you have COVID-19 and it tells you what precautions to take. Keep breastfeeding mamas, your milk is the best medicine for your babies!’ (C. LC2)

##### Encouraging messages and providing advices

Calming and comforting messages are important in crisis management and appeared as pieces of advice posted by mothers:

*‘*Keep on putting her on the breast. Switch sides and do skin to skin. Try to calm down in a dark room with the baby. The milk won’t disappear but the letdown is slowed down due to the stress. Don’t worry everything will be back to normal but u should keep offering the breast’ (C. M15).

Advices are also given by IBCLC:

‘Hello. Try to relax, close your eyes and think of your baby’ (C. LC1)

##### Emotional support sharing stories, feelings, attitudes and views, facing a challenging situation

Empathy and support appeared in quotes such as the one below, replying to stressed and traumatised mothers:

‘To all of you moms; from Spain I send you my love and support. I cannot imagine the shock you have to go through, maybe even grief, and I’m very afraid for the future of Lebanon. There are already many projects for donations. Luckily the world is responding (I heard of this through the “Lebanese in Madrid” page)’ (C LC3)

The post below is a mixture between sharing experience and showing the importance and positive impact of the group on breast-feeding journey, and, on the other hand, we could see the serious effect of Beirut’s explosion on breast-feeding.

‘After 6 o’clock from Beirut disaster everything is changed, coz my sadness i had a hard flu, the reason of my emotional and physical situation after Beirut explosion my kids were separated from me, now i am fine ((This change)) AFTER 6 o’clock, xxx breastfed 3 years day and night and today she is weaned. I am here in this group from 2014 when my breastfeeding journey began with my son and today it finished with my girl I had a passion to support nursing moms and this passion was a result for my work in breastmilk jewellery, I would thank all the group staff I will keep support moms in everything’ (P.M16)

#### Theme 2: community engagement

The posts on the Facebook groups showed how community members and healthcare professionals team up and volunteer to provide services to women in need for help. Several subthemes related to community engagement were identified:

##### Volunteering which draws people together to work for the benefit of others

Donations were the most common way of volunteering to help breast-feeding mothers. This subtheme was drawn from quotes such as

‘Why don’t we initiate a donation campaign of breast milk to babies in need in this crisis? I know some of you already do this so please let’s create a small group to do so, especially that moms who are bf cannot help on the field due to toxic substances.’ (P.M17)

*And,*

‘We have a group already for breastmilk donation: Human Milk 4 Human Babies – Lebanon’ (C. M18)

##### IBCLC and psychologists providing their services to the community

Mothers were anxious and stressed after the explosion. They were worried about their quality of milk, and many mothers faced difficulties in breast-feeding. However, IBCLC intervened to help:

‘Breastfeeding specialist xxxxx giving some quick information on F&Q’s of breastfeeding in Lebanon-right now and if stress, anxiety and grief can affect your supply’ (P.LC1).

We could tell that they were intervening and helping mothers by giving live sessions, or by offering consultations, that were beneficial to answer mothers’ concerns:

‘Hello all. mums who are suffering from difficulties in breastfeeding and especially after what happened in Lebanon…plz contact me to offer help’ (P.LC1).

As for psychologists, they were involved in the same way lactation consultants were and also offered free consultations to overcome the stressful period after Beirut’s explosion:

‘I’m a clinical psychologist you can call me if you want, i can help with the panic attack, maybe you won’t need medication’ (P.HC1)

#### Theme 3: material resources

Mothers needed material support such as pumping machines to keep on breast-feeding. Along with the economic crisis, the explosion affected mothers’ ability to breastfeed. Group members helped mothers who needed pumping machines in these critical moments by donating:

‘Hi all, I have a Medela Single Electric Breast Pump that was never used (not even once) and that I would like to donate to someone who really needs it. Please DM me if you want it and can pick it up from xxxxx this week. Hope you’re doing relatively ok in these difficult times’. (P. M19)

Milk donations were also needed in some cases. As another example of donation, some mothers intended to donate their own milk, as posted from this mother:

‘Hi Ladies I hope you are all safe. I have a quantity of breastmilk to donate, whoever is interested talk to me on messenger please’ (P. M20).

As a matter of fact, the explosion affected mothers’ ability to breastfeed due to stress or injury. So, to ensure breastmilk for these infants who could not receive their mother’s breastmilk, breastmilk donation started.

#### Theme 4: transformative potential

In order to build community resilience, stakeholders and active community members cooperate with each other to overcome barriers created in disrupted situations. As seen in the posts below, several social media groups addressed together, the problem of breastmilk availability.

##### Community network and cooperation with other organisations

Breast-feeding support groups on social media cooperated with each other for the support of the breast-feeding women community in Lebanon As we can read in this post:

‘We have a group already for breastmilk donation: Human Milk 4 Human Babies – Lebanon’

‘This community has pulled together so greatly before. You can also post in Human Milk 4 Human Babies – Lebanon’ (C. LC.2)

## Discussion

This is the first study in Lebanon that describes the role of social media in the promotion of breast-feeding. It identified the assets and challenges of breast-feeding in general and in moment of crisis specifically, and described the dynamics of community interaction on social media to encourage breast-feeding and to build community resilience. No previous study investigated the role of social media on building community resilience and on the empowerment of mothers for breast-feeding.

Identified facilitators and assets were re-enforced in moment of crisis and new assets emerged in addition to the existing ones to build community resilience post Beirut’s port explosion. These emerging assets were the role of IBCLC lactation consultants and psychologists who volunteered to help mothers facing problems, the emerging milk donation campaign which used to be a taboo and the cooperation between organisations.

As described in the literature, popularity of social media to discuss parenting issues and specially breast-feeding is increasing^(17)^. Although social media cannot catch the emotional or affective part of who writes on it, it is nowadays widely used to spread knowledge, messages and awareness due to its low cost and widespread^(31)^. This explains the high level of engagement between group members and admins in the studied Facebook breast-feeding support group. However, this interaction was not seen in other Lebanese Facebook breas-tfeeding support groups maybe due to the lower number of participants.

From the content analysis performed, we could conclude that challenges identified were similar to the ones reported in a previous systematic review done in low- and middle-income countries^(32)^, but the emerging economic crisis, pandemic and the Beirut’s port explosion added new challenges such as emotional stress affecting the perseverance to breastfeed and the lower financial capability that decreased the ability to buy breast-feeding gears and pumps. As for facilitators and assets that appeared mainly as support (informational, material and emotional), our findings are in line with studies identifying breast-feeding-related types of supports and assets on social media^(33)^. As it was also reported in previous studies, other topics discussed on the Facebook group are related to breast-feeding and health issues concerning mothers and infants during the breast-feeding period^(33,34)^.

The asset-based approach that was manifested as support is comparable to what was mentioned by Rippons S., Hopkins T.^(35)^. The first type of support reported here was emotional support. Members sharing their successful stories and sending encouraging messages helped mothers facing emotional stress. Then, informational support answered mother’s questions and concerns. This support was provided by IBCLC or group members by sharing articles, knowledge or advice. As it appeared in one of the studies done previously in Lebanon, mothers rely on online breast-feeding groups to receive information^(36)^. It is known that breast-feeding depends on both cognitive awareness and affective experiences. Hence, social media has an important role in transmitting tacit knowledge complementing the role of explicit knowledge, in improving breast-feeding practices and rates^(37)^. As for the material support, mothers offered to donate or sell breast-feeding gears at a reduced price. This important asset could improve breast-feeding rates with the inflated prices and economic hardship.

The value of these assets is due to the fact that they could increase the capability of individuals and communities to face a certain health issue^(35)^, especially in a vulnerable environment due to the economic crisis and political instability that Lebanon is facing.

As for the challenges facing Lebanese women to exclusively breastfeed and that could have played a role in early cessation of breast-feeding, we recognised the lack of support from peers, family and from healthcare professionals, lack of breast-feeding supportive policies and implementation, as well misconceptions and lack of knowledge. These findings are comparable with studies done in Lebanon^(7,38,39)^. Peers have a lot of misconceptions concerning EBF and could impose their opinion. As for paediatricians and healthcare staff that are highly influential on mothers, their knowledge is suboptimal as breast-feeding education is highly limited in medical school^(38)^. Another reported issue was the promotion of formula milk being a common practice^(39)^. All these factors could explain the perceived unsupportive behaviour of paediatricians towards EBF. As for breast-feeding policies and laws^(39)^, where maternity leave is limited, no BFHI is adopted, IBCLC are not part of healthcare system^(40)^ and marketing of formula milk is not prohibited, mothers are finding EBF challenging. Difficulties start with the unsupportive behavior of healthcare staff in maternity wards where they could offer water and glucose or formula milk instead of breastmilk, and where rooming in is not part of the hospitals’ policy^(7)^. Where the role of healthcare professional in breast-feeding was a barrier in our study, it was reported as facilitator in other countries^(41)^.

Our study was done amidst the biggest economic crisis in Lebanon since the Civil War. Added to that is the pandemic of COVID-19 and above all is the explosion of Beirut’s port on 4 August 2020^(20)^. In our analysis, we also attempted to focus on how community resources were involved in building community resilience following these circumstances that disrupted the country. Focusing on community resilience, we could retrieve access to social support including building a community network with communicating information and providing supportive environment, we could also retrieve community engagement where volunteers, IBCLC and healthcare professionals helped mothers to overcome stress and challenges in critical times by offering services and consultations free of charge. Resources were also present, and these helped mothers and infants who lack the ability to breastfeed or to buy pumping and breast-feeding gears. Lastly, there was the transformative potential where cooperation was built between different groups and organisations. All these were mentioned in studies investigating on how communities build resilience post crisis^(18)^. The increase in donations, offering help, giving emotional support and communicating scientific information were considered as assets necessary to promote and support breast-feeding through difficult time and to help mothers ‘bounce back’ from a stressful situation and event^(26,27)^. We have been able to assume that stressful living conditions in the country due to the economic crisis and pandemic made breast-feeding even more challenging. However, the support coming from mothers and lactation consultants provided a positive encouragement to deal with all these issues^(42)^.

Another important finding in this study is the emerging ‘donation’ as an important community asset. Donation of breastmilk and pumping machines and other gears is helpful to overcome the difficult financial situation and to help mothers who faced a decrease in milk supply after Beirut’s explosion. Many mothers were also injured so they wanted to receive breastmilk donations to give their infants instead of providing formula milk. This is how Facebook group for milk donations and milk donation campaign was created. Milk donations were completely not existent before Beirut’s explosion among mothers who do not know each other.

Milk banking is complicated and not common in Muslim countries. Milk donation is allowed in Islam when the donor is known. But, milk given from an anonymous mother is complicated as any child who breastfeeds more than five times from a donor will be considered as a brother/sister to the donor’s children and cannot marry each other^(43)^. Knowing that almost half the Lebanese population is Muslim, there are no milk bank in the country. However, after Beirut’s explosion, milk donations became more common and socially acceptable in order to provide breastmilk for infants due its several benefits.

### Limitation of the study

First of all, results should be read with caution as they correspond to the unique experience of social media groups and could not be generalised to other contexts. Our analysis was limited to one Facebook group in order to avoid duplication of data. In addition, one of the limitations of social media studies as seen also in this study is that the researchers are uninformed about the socio-economics and demographic information regarding the participants. Trying to minimise this, it was necessary to carefully select the social media group. Thus, researchers initially did a screening of the social media platforms of breast-feeding in Lebanon and then the social media group that includes the highest number of members was chosen to ensure the largest participation from different parts of the country and from different background. Then, researchers could not talk privately to members and ask to them how the group affected their breast-feeding experience, so there was a complete reliance on mothers’ comments praising the group and telling how helpful members were in the breast-feeding journey. Third, the quality of information posted was not evaluated.

Another limitation is the incomplete ability to assess through analysis of posts that contained tacit and explicit knowledge, the tacit knowledge and its role played in influencing breast-feeding practices. In future studies, it would be interesting to do a prospective study where we could collect members who gave birth and evaluate the duration of EBF, emphasising on how the group affected their breast-feeding journey.

### Implications of the study

Results withdrawn from this study have several policy implications to be taken into consideration for the effective promotion of breast-feeding in time of crisis. Social media should be recognised as an easily accessible asset. It is used to provide community support and to share knowledge and experience for effective management of breast-feeding. Furthermore, the important role of IBCLC in the promotion of breast-feeding should be reinforced for their positive contribution in the provision of professional advice to mothers. Having lactation consultants in maternity wards and planning after delivery follow-up with mothers could be examples of actions to engage them in services that promote breast-feeding.

## Conclusion

More strategies should be implemented to improve breast-feeding rates, added to the positive role of the community in empowering women to breastfeed by offering support, services and spreading knowledge. Since the role of the government and healthcare system is lagging, breast-feeding mothers are relying more on social media and community support. This asset-based approach has been playing a key role in breast-feeding promotion and building community resilience, during the COVID-19 pandemic, the economic crisis and after the catastrophe caused by Beirut Blast.
